# Pea Soup as a Substitute for Beef Tea

**Published:** 1889-12

**Authors:** 


### Pea-Soup as a Substitute for Beef-Tea.

Dr. Ris, of Kloten, Switzerland, says *The British Medical Journal* of September 28, emphatically recommends pea-soup as an excellent substitute for beef-tea for invalids, convalescents, and more especially for patients suffering from cancer of the stomach, or *diabetes mellitus.* Take peas, water, and sufficient amount of some vegetable suitable for soup, and one-half per cent. of carbonate of soda, and boil the whole until the peas are completely disintegrated; then let the soup stand until sedimentation is complete, and decant the fairly clear, thin fluid above the deposit. The product is stated to resemble a good meat-soup in its taste, to be at least equally digestible, and at the same time to surpass the very best meat-soup in nutritive value. The latter statement may appear surprising, but the author reminds us that peas (as well as beans or lentils, either of which may be used instead of peas) contain a considerable portion of legumen; that is, a vegetable albumin which is easily soluble in a faintly alkaline water, is not coagulated by heat, is easily absorbed, and equal to the albumin of eggs in its nutritiousness.—*Science,* October 18, 1889.

